# Deletion of SA β‐Gal+ cells using senolytics improves muscle regeneration in old mice

**DOI:** 10.1111/acel.13528

**Published:** 2021-12-13

**Authors:** Cory M. Dungan, Kevin A. Murach, Christopher J. Zdunek, Zuo Jian Tang, Georgia L. Nolt, Camille R. Brightwell, Zachary Hettinger, Davis A. Englund, Zheng Liu, Christopher S. Fry, Antonio Filareto, Michael Franti, Charlotte A. Peterson

**Affiliations:** ^1^ Department of Physical Therapy College of Health Sciences University of Kentucky Lexington Kentucky USA; ^2^ Sanders‐Brown Center on Aging University of Kentucky Lexington Kentucky USA; ^3^ The Center for Muscle Biology University of Kentucky Lexington Kentucky USA; ^4^ Computational Biology GCBDS Boehringer Ingelheim Pharmaceuticals Inc. Ridgefield Connecticut USA; ^5^ Department of Athletic Training and Clinical Nutrition College of Health Sciences University of Kentucky Lexington Kentucky USA; ^6^ Regenerative Medicine Boehringer Ingelheim Pharmaceuticals Inc. Ridgefield Connecticut USA; ^7^ Present address: Department of Health, Human Performance, and Recreation, and Cell and Molecular Biology Program University of Arkansas Fayetteville Arkansas USA

**Keywords:** regeneration, satellite cells, senescence, senolytics, skeletal muscle

## Abstract

Systemic deletion of senescent cells leads to robust improvements in cognitive, cardiovascular, and whole‐body metabolism, but their role in tissue reparative processes is incompletely understood. We hypothesized that senolytic drugs would enhance regeneration in aged skeletal muscle. Young (3 months) and old (20 months) male C57Bl/6J mice were administered the senolytics dasatinib (5 mg/kg) and quercetin (50 mg/kg) or vehicle bi‐weekly for 4 months. Tibialis anterior (TA) was then injected with 1.2% BaCl_2_ or PBS 7‐ or 28 days prior to euthanization. Senescence‐associated β‐Galactosidase positive (SA β‐Gal+) cell abundance was low in muscle from both young and old mice and increased similarly 7 days following injury in both age groups, with no effect of D+Q. Most SA β‐Gal+ cells were also CD11b+ in young and old mice 7‐ and 14 days following injury, suggesting they are infiltrating immune cells. By 14 days, SA β‐Gal+/CD11b+ cells from old mice expressed senescence genes, whereas those from young mice expressed higher levels of genes characteristic of anti‐inflammatory macrophages. SA β‐Gal+ cells remained elevated in old compared to young mice 28 days following injury, which were reduced by D+Q only in the old mice. In D+Q‐treated old mice, muscle regenerated following injury to a greater extent compared to vehicle‐treated old mice, having larger fiber cross‐sectional area after 28 days. Conversely, D+Q blunted regeneration in young mice. *In vitro* experiments suggested D+Q directly improve myogenic progenitor cell proliferation. Enhanced physical function and improved muscle regeneration demonstrate that senolytics have beneficial effects only in old mice.

## Graphical abstract

Senolytics improve muscle regeneration in old mice, while providing no benefit in young mice. Old mice display more satellite cells, reduced inflammation, a lower senescent cell burden, and increased gene expression of bioenergetic pathways relative to untreated old mice.

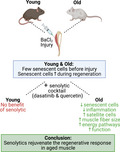

## 1 INTRODUCTION

One hallmark indicator of organismal aging is the accumulation of senescent cells; cells that have permanently exited the cell cycle due to factors such as replicative exhaustion, unrepaired DNA damage, or oncogene stress (reviewed in Gorgoulis et al. (Gorgoulis et al., 2019)). The accumulation of these cells has been linked to altered metabolism (Ademowo et al., 2017; Palmer et al., 2019), reduced cognitive function (Ogrodnik et al., 2019; Zhang, Swarts, et al., 2019), lower physical function (Baker et al., 2016; Xu et al., 2018), and shortened lifespan (Baker et al., 2016; Xu et al., 2018) largely through the production of the senescence‐associated secretory phenotype (SASP). The SASP is comprised of a diverse panel of chemokines, inflammatory cytokines, proteases, growth factors, and hundreds of other molecules (Basisty et al., 2020; Coppe et al., 2010) that negatively affect the surrounding environment, causing inflammation and induction of senescence in neighboring cells. Genetic (Baker et al., 2011, 2016; Palmer et al., 2019) and pharmacologic (Ogrodnik et al., 2019; Xu et al., 2018; Zhang, Swarts, et al., 2019) clearance of senescent cells throughout the body improves all of the aforementioned outcomes, reducing the burden of the SASP on whole‐body metabolic and physiologic homeostasis.

Although senescent cell accumulation is generally considered maladaptive, senescent cells are essential to the reparative process in regenerating tissues and wound healing (Da Silva‐Alvarez et al., 2020; Demaria et al., 2014; Sarig et al., 2019). Skeletal muscle is especially adept at repair, as muscle is completely regenerated 28 days following barium chloride (BaCl_2_) injury in the mouse (Hardy et al., 2016). With aging, regeneration is impaired, resulting in smaller muscles and muscle fibers when compared to young mice following BaCl_2_ injury (Blanc et al., 2020; Endo et al., 2020; Lee et al., 2013). There are various contributing factors to compromised regeneration with age including fewer muscle stem cells (satellite cells) (Dungan et al., 2020; Shefer et al., 2006, 2010), reduced satellite cell function (Pietrangelo et al., 2009), and altered muscle environment with higher expression of anti‐regenerative factors such as GDF11 (Egerman et al., 2015); however, the accumulation of senescent cells, identified as senescence‐associated β‐Galactosidase positive (SA β‐Gal+), could also play a role. In young adult animals, SA β‐Gal+ senescent cells accumulate early following damage in muscle (Cazin et al., 2017; Chiche et al., 2017; Doan et al., 2020; He et al., 2019; Sarig et al., 2019), but are reduced as the tissue regenerates (He et al., 2019; Sarig et al., 2019). Therefore, this work was designed to test the hypothesis that aberrant accumulation of senescent cells may negatively impact muscle regeneration during aging.

Senescent cell killing compounds, termed senolytics, are a new category of pharmaceuticals aimed at combating age‐associated diseases, systemically clearing senescent cells by removing the brakes on senescent cell anti‐apoptotic pathways (SCAPs) (Zhu et al., 2015, 2016). The combination of dasatinib and quercetin is especially effective at removing senescent cells in a host of tissues (Chu et al., 2020; Li et al., 2021; Palmer et al., 2019; Zhang, Swarts, et al., 2019; Zhu et al., 2015), all while improving physical function and extending lifespan (Xu et al., 2018). Although there are conflicting reports on the abundance of senescent cells in resting muscle from old mice (Dungan et al., 2020; Silva et al., 2019), recent studies have demonstrated that 10 days following cardiotoxin injury to muscle, senolytic drugs elevate satellite cell numbers and are associated with a greater number of large myofibers (Doan et al., 2020), in addition to reducing the number of SA β‐Gal+ cells in damaged muscle tissue (Chiche et al., 2017). The goals of this study were to determine (1) the origin of senescent cells in injured muscle, (2) if injury causes a sustained elevation in senescent cells in muscle from aged mice, and (3) if removing senescent cells using senolytics (dasatinib and quercetin) enhances muscle regeneration in old mice over a time course of 28 days.

## 2 MATERIALS AND METHODS

### 2.1 Animals

Young (3 months; *n* = 44) and old (20 months; *n* = 38) male C57Bl/6J mice were obtained from Jackson Labs (Bar Harbor, ME). All animal procedures were approved by the IACUC of the University of Kentucky. Mice were housed in a temperature and humidity‐controlled room, maintained on a 14:10‐h light‐dark cycle, and food and water were provided *ad libitum*. Mice were euthanized via exsanguination under isoflurane anesthesia, followed by cervical dislocation, and were fasted 6 h prior to euthanization. The study originally included *n* = 40 old mice, but two old mice died before the end of the study. During tissue removal, the TA was weighed and then cut lengthwise; ~1/3 of the muscle was snap frozen in LN2 and stored at −80°C for RNA isolation and ~2/3 of the muscle was covered in OCT, frozen in LN2‐cooled isopentane, and stored at −80°C for immunohistochemistry (IHC).

### 2.2 Senolytics

**FIGURE 1:**
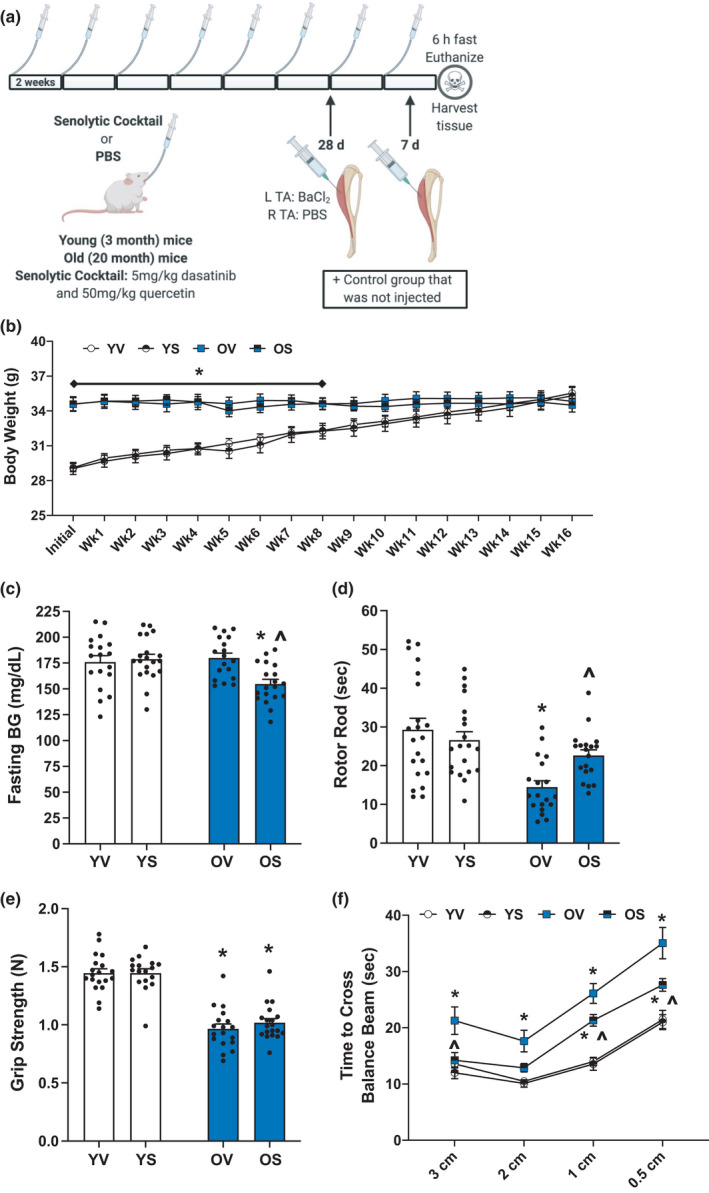
Senolytics lower blood glucose and improve physical function in old mice. (a) Study design schematic. (b) Weekly body weight for YV (white circles), YS (black and white circles), OV (blue squares), and OS (black and blue squares) mice. (c) Fasting blood glucose measurements in mg/dl. (d) Average time spent on the rotor rod in seconds. (e) Average forearm grip strength in Newtons. (f) Time to cross a 3 cm, 2 cm, 1 cm, and 0.5 cm wide balance beam for YV (white circles), YS (black and white circles), OV (blue squares), and OS (black and blue squares) mice. Error bars indicate −/+ the standard error of the mean. *p < 0.05 between young and old for an individual treatment group (YV vs. OV; YS vs. OS). ^*p* < 0.05 between vehicle and senolytics for a given age group (YV vs. YS; OV vs. OS). *n* = 17–20/group

Mice were administered a senolytic cocktail containing 5 mg/kg dasatinib (D‐3307, LC Labs) and 50 mg/kg quercetin (Q4951, Sigma‐Aldrich) as described by Xu et al. (Xu et al., 2018). Briefly, 7.5 mg of dasatinib and 75 mg of quercetin were dissolved in 5 ml of 10% polyethylene glycol 400 (PEG 400; 202398, Sigma‐Aldrich). We chose this volume of PEG 400 because it allowed us to gavage a 30–45 gram mouse with 100–150 μl of D+Q, which is less than the approximate stomach volume of 400 μl in adult mice (McConnell et al., 2008). Mice were gavaged bi‐weekly for 4 months (Figure 1a) with D+Q (*n* = 20 young and *n* = 19 old) or vehicle (*n* = 20 young and *n* = 19 old; 10% PEG 400) using 20‐gauge disposable polypropylene feeding tubes (FTP‐20‐30, Instech, Plymouth Meeting, PA). These mice were then divided into 3 groups of *n* = 6–7/group.

### 2.3 BaCl_2_ injections

The left tibialis anterior (TA) muscle was injected with 1.2% BaCl_2_ (342920, Sigma‐Aldrich) in 5 locations with 10 μl of 1.2% BaCl_2_ injected at each location (50 μl total volume). The right TA served as the internal control muscle and was injected with phosphate‐buffered saline (PBS) in the same manner. Mice were euthanized 7‐ (*n* = 6–7/group) and 28 days (*n* = 5–6/group) post‐injection. Subsets of mice were killed at 7‐ and 14 days for isolation of SA β‐Gal+ cells (*n* = 8 young and *n* = 8 old). Groups of mice (*n* = 6/group) served as non‐injected controls. Originally, the 28‐day BaCl_2_ groups of mice included *n* = 7–8/group; however, 2 mice were excluded from each group due to inadequate administration of BaCl_2_, as determined by a lack of central myonuclei throughout the muscle.

### 2.4 Blood glucose

Mice were fasted 6 h prior to euthanization. Mice were anesthetized under 3% isoflurane, and a small nick was made at the distal end of the tail. One drop of blood was placed on a blood glucose test strip and measured with a glucometer (Clarity BG1000, Clarity Diagnostics). If the blood glucose value was below 100 mg/dl or above 250 mg/dl, it was remeasured.

### 2.5 Physical function tests

Physical function tests were performed using established protocols through the University of Kentucky Rodent Behavior Core. To test balance and coordination, rotor rod and balance beam tests were performed. Physical function was assessed using a grip strength test. The rotor rod test was performed using a 4‐lane SDI ROTOR ROD System (San Diego Instruments) with a 45.7 cm fall height. Rotor rod data were collected using ROTOR ROD software (San Diego Instruments). The balance beam test was performed using a homemade device with a fall height of 15 cm and beam widths of 3 cm, 2 cm, 1 cm, and 0.5 cm. Grip strength was performed using Columbus Instruments Grip Strength Meter (Columbus Instruments). For all tests, lighting was set at approximately 75 lux and air temperature was maintained at approximately 25°C.

#### 2.5.1 Rotor rod

Mice were acclimated to the rotor rod for 60 s at 4 rpm. If a mouse fell off during that 1‐min acclimation period, they were immediately placed back on. Thirty minutes after the training session, mice were placed on the rotor rod with a ramp‐up profile set at 0–40 rpm and 0–300 s. No mice were able to stay on the rotor rod for 300 s. Mice performed 3 consecutive trials, and data are shown as the average value among the 3 trials.

#### 2.5.2 Balance beam

Mice were acclimated by allowing them to traverse back and forth on the 3 cm balance beam for 60 s. After a 30‐min rest, mice were given up to 60 s to cross each beam. Mice started on a 3 cm wide beam and progressively moved to a 2 cm, 1 cm, and 0.5 cm beam. The time to cross and number of foot slips were recorded.

#### 2.5.3 Grip strength

Forearm grip strength was assessed over 3 consecutive trials. Mice were gently picked up by their tail and held in front of the grip strength meter. Once the mouse grabbed the grip strength meter, they were quickly, but gently, pulled backwards to measure the forearm grip strength. Three consecutive trials were performed, and data are shown as the average of the 3 trials.

### 2.6 5‐Dodecanoylaminofluorescein Di‐β‐D‐galactopyranoside (C_12_FDG), CD11b labeling, and fluorescence‐activated cell sorting (FACS)

TA muscles from 8 untreated young (4 months) and 8 untreated old (24 months) mice were injected with 1.2% BaCl_2_ 7‐ (*n* = 4/age group) or 14‐ (*n* = 4/age group) days prior to euthanization as described above. These muscles were then pooled for this experiment. For FACS, muscles were processed according to a protocol adapted from the Rando laboratory (Liu et al., 2015) that has previously been used by our laboratory (Murach et al., 2020). In brief, minced muscle was digested with collagenase in sorting media (10% normal horse serum/Hams F‐10/penicillin‐streptomycin), then collagenase and dispase in sorting media at 37°C. Cells were aspirated with a 20‐gauge needle, strained through 40 µm filters, pelleted, and re‐suspended in 500 µl of sorting media. To identify senescent cells, we used the ImaGene Green fluorescent labeling kit (I2904, Invitrogen), which contains the β‐galactosidase substrate, C_12_FDG, in a manner consistent with others (Cai et al., 2020; Debacq‐Chainiaux et al., 2009). Cells were incubated with 5 µl chloroquine for 30 min at 37°C to reduce the labeling of endogenous β‐galactosidase in healthy cells with C_12_FDG, then the cell pellet was spun and washed. Cells were re‐suspended in 1 ml of sorting buffer and incubated with 1.75 µl of C_12_FDG and 5 µl of CD11b PE‐conjugated primary antibody (101207, BioLegend) for 1 h at 37°C, then the cell pellet was washed again. Finally, the cells were re‐suspended in 1 ml of sorting buffer and treated with 20 µl of PETG and propidium iodide. Since chloroquine inhibits endogenous but not SA β‐Gal (Poot & Arttamangkul, 1997; Tietz et al., 1990), aliquots of SA β‐Gal negative cells that were treated with and without chloroquine were used to determine gating (Figures S2a,b). Cells were sorted using an iCyt FACS machine (Sony Biotechnology).

Following FACS, cells were pelleted at 500 RPM onto slides using a Cytospin™ 4 (Thermo Scientific), or prepared for RNA isolation. Slides were then dried for 15 min and incubated in DAPI (1:10,000; D1306, Invitrogen), cover slipped in PBS and glycerol (1:1) and imaged to visualize C_12_FDG+ and CD11b+ cells. Cells were also stained for senescence‐associated β‐Galactosidase (SA β‐Gal).

### 2.7 Myogenic progenitor cell (MPC) treatment with senolytics and *in vitro* proliferation assay

Myogenic progenitor cells from young (4 months) and aged (24 months) mice were used for experimentation. On ECM‐coated 6‐well plates, 20 × 10^3^ MPCs from young and aged mice were treated with 250 nM dasatinib and 50 µm quercetin in growth media for 24 h; cells without senolytics served as controls. After 24 h, cells were washed and treated with 5 µm EdU (E10187, Invitrogen) in growth media for another 24 h to quantify DNA synthesis. After 24 h, growth media was removed, and cells were washed in PBS and fixed in 4% paraformaldehyde (PFA) for 10 min. EdU was detected using Click It chemistry adapted from Kirby et al. (Kirby et al., 2016), followed by counterstaining with DAPI.

### 2.8 Immunohistochemistry (IHC)

All IHC was performed on fresh frozen muscle cross‐sections. Muscle tissue was removed from −80°C storage and placed into a cryostat (HM525 NX; Thermo Fisher) set at −24°C. After the tissue warmed to −24°C, 8 μm sections were cut and allowed to dry for at least 1 h before IHC analysis or slides were stored at −80°C for future experiments. For all IHC experiments, no primary antibody controls were used to determine background signal. Data from PBS‐injected TAs (7 and 28 days) were merged into one group.

#### 2.8.1 Embryonic myosin heavy chain (eMyHC)/Laminin/DAPI

Sections were blocked in 2% bovine serum albumin (BSA) plus M.o.M. (MKB‐2213, Vector Labs) for 60 min. Sections were then incubated in primary antibodies against eMyHC (supernatant 1:20; F1.652, Developmental Studies Hybridoma Bank) and laminin (1:200; L9393, Sigma‐Aldrich) diluted in 2% BSA for 90 min, washed in PBS, and incubated in secondary antibodies against Ms IgG1 AF594 (1:200; A‐21125, Invitrogen) and Rb IgG AF488 (1:200; A‐11008, Invitrogen) diluted in PBS for 60 min. Sections were washed, incubated in DAPI (1:10,000; D1306, Invitrogen) for 15 min, washed in PBS, and cover slipped using PBS and glycerol (1:1).

#### 2.8.2 Pax7/Laminin/DAPI

Sections were fixed in 4% PFA for 10 min, washed in PBS, incubated in 3% H_2_O_2_ for 10 min, and washed again in PBS. Heat‐mediated antigen retrieval was performed in 10 mM sodium citrate pH 6.5. Sections were placed in 65°C 10 mM sodium citrate and then gradually heated to 92°C (~20 min) using a water bath. Once at 92°C, sections remained in the 10 mM sodium citrate for 12 min, then allowed to cool for 60 min. Sections were then washed in PBS, blocked in 2% BSA plus M.o.M. (Vector Labs) for 60 min, washed in PBS again, and incubated in primary antibodies against Pax7 (concentrate 1:100; PAX7, Developmental Studies Hybridoma Bank) and laminin (1:200; L9393, Sigma‐Aldrich) diluted in 2% BSA overnight. The following day, sections were washed in PBS, incubated in a secondary antibody against Ms IgG1 Biotin (1:1000; 115‐065‐205; Jackson ImmunoResearch) in 2% BSA for 90 min, washed in PBS, and incubated in secondary antibodies streptavidin horseradish peroxidase (1:500; S‐911, Invitrogen) and Rb IgG AF488 (1:200; A‐11008, Invitrogen) diluted in PBS for 75 min. Following another PBS wash, sections were incubated in TSA AF594 (1:500; B40957, Invitrogen) diluted in DAPI staining solution (1:10,000; D1306, Invitrogen) for 15 min, washed in PBS, and cover slipped in PBS and glycerol (1:1).

### 2.9 Senescence‐associated beta‐galactosidase (SA β‐Gal)

Senescence‐associated beta‐galactosidase was adapted from a previously published protocol by our laboratory (Dungan et al., 2020). Briefly, slides containing muscle sections and cells were fixed in 0.5% glutaraldehyde for 5 min at room temperature and washed in PBS. After washing, sections were incubated in freshly made staining solution that contained: 1 mg/ml X‐gal in DMF, 5 mM potassium ferrocyanide, 5 mM potassium ferricyanide, 5 M sodium chloride, 1 M magnesium chloride, and 0.2 M citric acid/Na phosphate buffer pH 6.0 ± 0.05. Muscle sections were incubated in staining solution for 72 h at 37°C in a dark hybridization oven, with fresh solution added every 24 h. Afterward, sections were washed in PBS for up to 24 h to remove salt crystals (this does not affect staining). Sections were then post‐fixed in 0.5% glutaraldehyde for 10 min, washed in PBS, briefly stained (1 min in each) for hematoxylin and eosin to identify fibers and label nuclei, cleared, and then cover slipped using Cytoseal 60 (8310‐16, Thermo Scientific) permanent mounting medium.

### 2.10 Automated muscle fiber analysis

Average myofiber cross‐sectional area (CSA) quantification was performed using MyoVision (Wen et al., 2018). eMyHC and laminin labeled images were used to automatically determine CSA of eMyHC+ and eMyHC‐ fibers by MyoVision, with CSAs below 100 µm^2^ and above 6000 µm^2^ excluded from the analysis. On average, greater than 1000 fibers were analyzed for each muscle to quantify myofiber CSA. For all images, parts of the cross‐section that appeared to be folded or damaged during cryosectioning, along with regions that were not damaged by BaCl_2_, as determined by a lack of eMyHC expression at 7 days or central nuclei at 7 and 14 days (example in Figure S1a), were manually excluded from the analysis.

### 2.11 RNA isolation

Frozen muscle and freshly isolated cells were placed into 1.5 ml Safe‐Lock Eppendorf tubes (022363212, Eppendorf) containing QIAzol Lysis Reagent (79306, Qiagen) and homogenized using 2.0 mm zirconium oxide beads (ZROB20, Next Advance) in a Bullet Blender bead homogenizer (BBY24M, Next Advance) at max speed until the muscle was completely disintegrated (3–4, 1‐min cycles). RNA was isolated using the RNeasy Plus Mini Prep kit (74136, Qiagen); however, we utilized the Qiagen guanidine/phenol‐based protocol instead of the protocol associated with this kit. RNA concentration was determined via NanoDrop (NanoDrop 2000, Thermo Fisher), and RNA integrity numbers (RINs) were quantified using a 2100 Bioanalyzer (Agilent). All RINs were >8.0 with an average value of 9.1.

### 2.12 Statistical analysis

For analyses comparing four groups (young vehicle, young senolytic, old vehicle, old senolytic) at any given time point (PBS‐injected, 7D‐post‐BaCl_2_, 28D‐post‐BaCl_2_), a two‐way ANOVA was performed with significance set at *p* < 0.05. If significance was detected, a Holm‐Sidak post hoc test was used to identify significant comparisons between age (young vs. old) and treatment (vehicle vs. senolytic) groups. A repeated measures two‐way ANOVA was used to determine significance for body weight each week over the course of the study. Statistics were performed using Prism 9 software for Mac (GraphPad Software).

## 3 RESULTS

### 3.1 D+Q‐treated old mice have improved physical function

Young and old C57Bl/6J mice were treated with a senolytic cocktail of D+Q bi‐weekly for 4 months (Figure 1a), so the mice were 7 months and 24 months of age, respectively, at the time of functional testing and tissue collection for immunohistochemical analyses. This treatment regimen has previously been shown to improve physical function in old mice (Xu et al., 2018). There was no effect of senolytics on body mass (Figure 1b); however, lower fasting blood glucose was observed in old senolytic‐treated (OS) mice compared to old vehicle‐treated mice (OV, Figure 1c). Physical function assessments showed improvements in rotor rod (Figure 1d) and balance beam (Figure 1f) in OS mice, whereas grip strength was unchanged and remained significantly lower than young mice (Figure 1e). For all outcomes in Figure 1, there was no significant difference between young vehicle‐treated (YV) and young senolytic‐treated (YS) groups.

### 3.2 D+Q does not have a substantial effect on systemic inflammation markers in serum

We utilized a multiplex ELISA for 36 inflammatory markers, including common SASP markers such as IL‐1α, IL‐1β, IL‐6, and MCP‐1, to determine if D+Q reduce markers of systemic inflammation. Of the 36 proteins in the ELISA, only 14 were sufficiently abundant in serum for analysis. Of the 14 proteins, only MCP‐3 abundance was affected by D+Q; it was lower in YS mice compared to YV with no effect in the old mice (Table S1). Thus, there does not appear to be a large effect of D+Q on the markers of systemic inflammation included on the panel.

### 3.3 D+Q preferentially reduces senescence‐associated β‐Galactosidase (SA β‐Gal+) cell burden in old mice 28 days following injury

**FIGURE 2:**
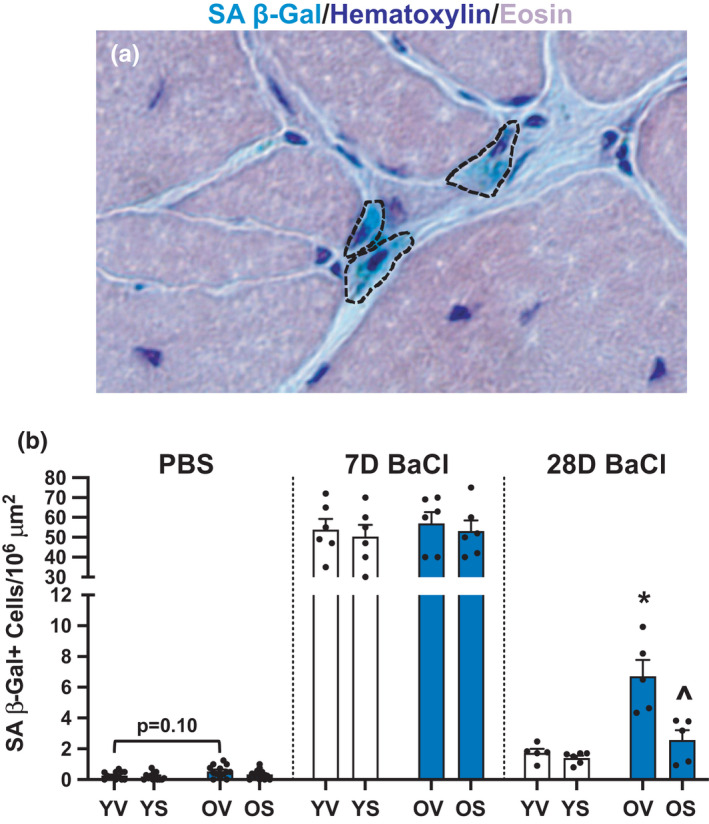
Senolytics lower SA β‐Gal+ cell burden 28 days following BaCl_2_ injury. (a) Representative image of SA β‐Gal (blue staining outlined in black), hematoxylin (dark purple nuclei), and eosin (light purple cytoplasm). (b) SA β‐Gal+ cell abundance per area in YV, YS, OV, and OS mice. Young mice are labeled with white bars and old mice are labeled with blue bars. Error bars indicate −/+ the standard error of the mean. *p < 0.05 between young and old for an individual treatment group (YV vs. OV; YS vs. OS). ^*p* < 0.05 between vehicle and senolytics for a given age group (YV vs. YS; OV vs. OS). *n* = 12–14/group for PBS‐injected mice. *n* = 5–6/group for 7‐ and 28‐day BaCl_2_‐injected mice

To test the effect of D+Q on muscle regeneration, the tibialis anterior (TA) muscles of YV, YS, OV, and OS mice were injected with either PBS or BaCl_2_ and harvested after 7‐ or 28 days (Figure 1a). Using SA β‐Gal staining (representative images, Figure 2a and Figure S1b), we found that SA β‐Gal+ cells were of very low abundance in PBS‐injected muscle regardless of age (Figure 2b); although there was a trend (*p* = 0.10) to have more senescent cells in PBS‐injected old TA muscles (Figure 2b). This could be due to the physical trauma from the needle injection, as SA β‐Gal+ cells were not detected in injection‐naïve muscle in either age group (data not shown). Following BaCl_2_ injection, SA β‐Gal+ cells were present throughout the damaged muscle area, identified by embryonic myosin heavy chain‐expressing (eMyHC+) fibers (Figure S1a), with small areas spared from damage largely free of SA β‐Gal+ cells (Figure S1b). There was a large increase in SA β‐Gal+ cells at 7 days (~0.2 per 10^6^ μm^2^ in PBS‐injected compared to ~50 per 10^6^ μm^2^ in BaCl_2_ injected), with no difference in the abundance of SA β‐Gal+ cells between age groups at this time point. At 28 days post‐injury, OV mice had significantly more SA β‐Gal+ cells than YV mice (Figure 2b). There was no effect of D+Q 7 days following injury in young or old mice (Figure 2b); however, there were significantly fewer SA β‐Gal+ cells in OS mice 28 days after injury compared to OV (Figure 2b).

### 3.4 SA β‐Gal+ cells that emerge during muscle regeneration are primarily immune cells

**FIGURE 3:**
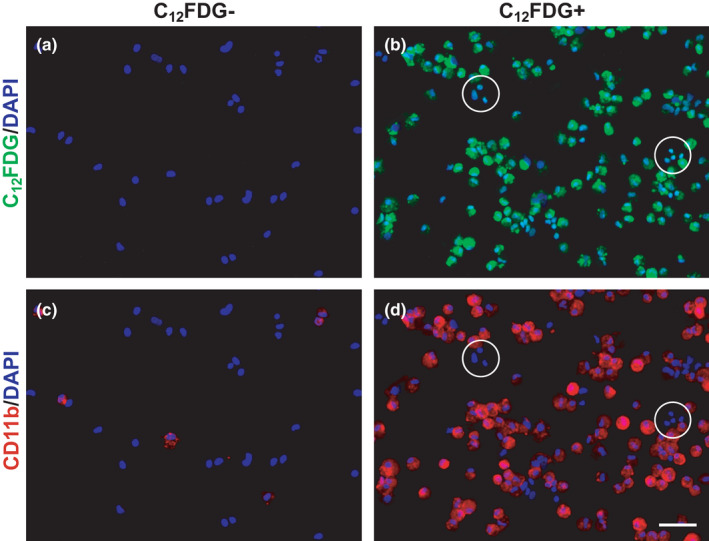
Most C_12_FDG+ cells are CD11b+ suggesting they are infiltrating macrophages. Muscles from old mice 14 days following injury were dissociated and cells sorted as described in Figure S2. Representative images of isolated (a) C_12_FDG− and (b) C_12_FDG+ cells. Cells were immunoreacted with a CD11b antibody (red) shown in (c) C_12_FDG−/CD11b+, and (d) C_12_FDG+/CD11b+ cells. White circles in b and d indicate rare C_12_FDG+/CD11b− cells. Cells were also labeled with DAPI (blue) to visualize nuclei. *n* = 1 pooled sample of 4 TAs for each group. Scale bar equals 100 μm

Macrophage infiltration is necessary for muscle repair (Liu et al., 2017; Segawa et al., 2008; Wang et al., 2014) and reports show that macrophages can express SA β‐Gal (Childs et al., 2016): macrophages polarizing from M1 to M2 display high expression of senescence markers p16 and SA β‐Gal, which are then reduced in fully differentiated M2 macrophages (Hall et al., 2016, 2017). Therefore, we hypothesized that the large number of SA β‐Gal+ cells 7 days following BaCl_2_ were likely infiltrating macrophages in muscle from both young and old mice. We further hypothesized that these cells are normally cleared following muscle repair, or complete transition to an M2‐like phenotype in young muscle, whereas in old muscle, the macrophages become senescent over time. We used C_12_FDG to label SA β‐Gal+ cells fluorescently green, in the presence of chloroquine, which raises lysosomal pH of the cell to limit the labeling of non‐senescent cells by reducing endogenous, but not SA β‐Gal enzyme activity, as shown by fluorescence‐activated cell sorting (FACS) (Figures S2a,b) (Cahu & Sola, 2013; Nogueira‐Recalde et al., 2019; Poot & Arttamangkul, 1997; Tietz et al., 1990). Cells were also labeled using an antibody against the pan macrophage marker, CD11b, and GFP+ and GFP− cells were isolated by FACS at 7‐ and 14 days post‐BaCl_2_ injury (Figures S2c–e). Immunocytochemistry showed that C_12_FDG− cells (Figure 3a) were also CD11b− (Figure 3c). Greater than 90% of the C_12_FDG+ cells (Figure 3b) were CD11b+ (Figure 3d). The white circles in Figure 3b,d indicate the less than 10% of C_12_FDG+ cells are CD11b‐. Staining for SA β‐Gal of sorted cells confirmed that the GFP+ cells were SA β‐Gal+ positive (Figures S2f,g).

To determine if the phenotype of the CD11b+ cells differed in young and old mice following injury, FACS‐isolated cells from young and old mice (*n* = 4 in each age group) were pooled and profiled using low‐input RNA sequencing (RNA‐seq) due to the relatively low amount of RNA contained within cells. We were unable to isolate sufficient cells at the 28‐day time point so RNA was isolated from cells at 7‐ and 14 days post‐injury. Comparing C_12_FDG+/CD11b+ from muscle of young and old mice, there were relatively few differentially expressed genes 7 days post‐BaCl_2_ injury (DEGs; 52 genes); however, some of the more highly expressed DEGs in cells from old compared to young mice included chemokines *Ccl8*, *Ccl12*, *Cxcl9*, and *Cxcl10*, as well as *Neat1*, an activator of the macrophage inflammasome (Zhang, Cao, et al., 2019), and *Klf4*, a transcription factor that upregulates the senescence marker and cell cycle inhibitory protein, p21 (Gamper et al., 2012; Xu et al., 2016) (File S1). At 14 days, there were fewer C_12_FDG+/CD11b+ cells in regenerating muscle from young (9.9% of total cell count) compared to old mice (11.8% of total cell count), suggestive of clearance from the muscle by this time point preferentially in muscle from young mice, and there were hundreds of DEGs between cells from young and old mice. In the C_12_FDG+/CD11b+ cells that were present in old mice at 14 days, there was significant upregulation of genes encoding chemokines and inflammatory cytokines (741 genes), including well‐accepted components of the SASP (i.e., *Ccl2, Il‐1a, Il‐1b, Tnf*, and *Gdf15*), in addition to higher expression of *Cdkn1a*, encoding p21 (File S1). In C_12_FDG+/CD11b+ cells from young mice, there were few inflammatory cytokines expressed at 14 days. On the other hand, expression of *Adamts1* and *Pdgfb* was higher in cells from young compared to old mice, factors secreted by M2 macrophages shown to promote muscle stem cell activation (Doumit et al., 1993; Du et al., 2017) (File S1). Together, our data suggest that SA β‐Gal+ cells are CD11b+ macrophages at 7 days post‐injury in both young and old mice, whereas by 14 days these cells from old mice display a SASP and upregulation of p21, indicating they are becoming senescent.

### 3.5 Muscle regeneration is enhanced in D+Q‐treated old but not young mice

**FIGURE 4:**
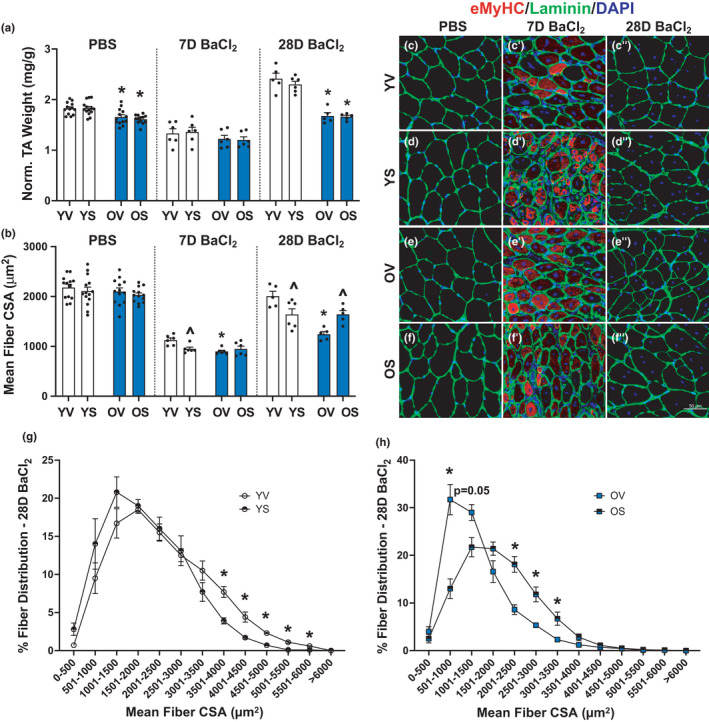
Differential effects of senolytics on muscle fiber size during regeneration in young and old mice. Young mice are labeled with white bars and old mice are labeled with blue bars. (a) TA weight normalized to body weight. (b) Mean fiber CSA. (c–f) Representative images of immunohistochemical staining for eMyHC (red), laminin (green), and DAPI (blue) in PBS‐injected, (c’–f’) 7 days following BaCl_2_ injection, and (c’’–f’’) 28 days following BaCl_2_ injection. (g) Fiber size distribution for YV and YS groups. (h) Fiber size distribution for OV and OS groups. Error bars indicate −/+ the standard error of the mean. *p < 0.05 between young and old for an individual treatment group (YV vs. OV; YS vs. OS). ^*p* < 0.05 between vehicle and senolytics for a given age group (YV vs. YS; OV vs. OS). *n* = 12–14/group for PBS‐injected mice. *n* = 5–6/group for 7‐ and 28‐day BaCl_2_‐injected mice. Scale bar equals 50 μm

To determine if clearance of senescent cells influenced TA muscle regeneration, muscles of YV, YS, OV, and OS mice injected with either PBS or BaCl_2_ were harvested for IHC after 7‐ or 28 days. Normalized TA mass was reduced 7 days post‐injury but was not significantly different between YV and OV mice (Figure 4a). Fiber CSA was nearly 50% lower at 7 days, with OV mice having a smaller mean fiber CSA compared to YV (Figure 4b). The greater reduction in fiber CSA compared to muscle mass 7 days post‐injury is likely due to edema and infiltrating immune cells. Muscle regeneration was clearly ongoing as eMyHC‐expressing fibers were apparent in both age groups (representative images shown in Figure 4c–f). At the 28‐day timepoint, YV mice had larger muscles than OV (Figure 4a), in addition to having larger fiber CSA (Figure 4b). Although fiber CSA was similar between uninjured and injured muscle in young mice after 28 days, BaCl_2_‐injected muscles from YV mice were larger than PBS‐injected muscles. This is likely due to an increase in total fiber number following BaCl_2_ injection (Figure S3a–c). Fibers in OV mice were smaller 28 days following BaCl_2_ injection compared to OV following PBS injection (Figure 4b); however, muscle mass was not different between those groups (Figure 4a). Similar to the young, this can be attributed to a difference in total fiber number, as OV 28‐day BaCl_2_‐injected mice had more fibers than OV PBS injected (Figure S3c).

D+Q had different effects on muscle regeneration in young compared to old mice. In young mice, fiber CSA of YS mice was smaller when compared to YV at both 7‐ and 28 days post‐injury (Figure 4b), which corresponded to a leftward shift in the histogram of fiber size distribution in YS and lower abundance of larger fibers compared to YV at 28 days (Figure 4g). At 7 days post‐injury, YV had fewer small regenerating fibers, which corresponded with fewer small eMyHC+ fibers than YS (Figure S3d,e). There was no effect of D+Q on mean fiber CSA (Figure 4b) or mean fiber size distribution in old mice 7 days following injection (Figure S3f), but a tertiary analysis of fiber size distribution of nascent eMyHC+ fibers revealed that OS mice had a great proportion of larger regenerating fibers than OV (Figure S3g). After 28 days, fiber CSA in OS mice was significantly larger than OV mice (Figure 4b), corresponding to a rightward shift to a larger fiber size distribution in OS compared to OV mice (Figure 4h). Thus, D+Q appear to augment muscle regeneration in old mice and impair regeneration in young mice.

Because senescent cells have been associated with tissue fibrosis in the lung (Citrin et al., 2013; Minagawa et al., 2011; Schafer et al., 2017), total fibrous collagen content, as well as the content of loosely and tightly packed collagen, were assessed using Sirius red staining followed by visible light (Figure S4a) and polarized light (Figure S4c) imaging, respectively, quantified in Figure S4b,d. Overall collagen abundance was unchanged by D+Q, along with no effect of age (Figure S4b). Collagen organization was assessed in a manner consistent with Smith et al. (Smith & Barton, 2014), and we observed no difference in the relative organization of collagen (Figure S4d) between any group 28 days following BaCl_2_ injury.

### 3.6 Satellite cell abundance and activation is higher with D+Q in old mice

**FIGURE 5:**
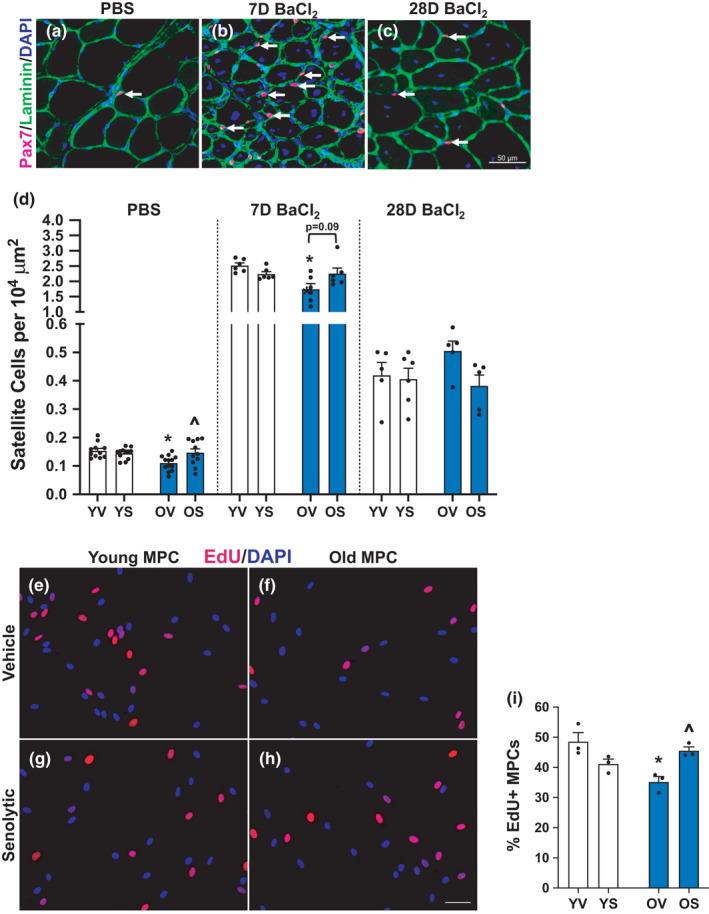
Senolytics increase satellite cell abundance in old mice. Young mice are labeled with white bars and old mice are labeled with blue bars. Representative images of immunohistochemical staining for Pax7 (pink), laminin (green), and DAPI (blue) from (a) PBS‐injected, (b) 7 days following BaCl_2_ injection, and (c) 28 days following BaCl_2_ injection. (d) Satellite cell abundance per area. Error bars indicate −/+ the standard error of the mean. Scale bar equals 50 μm. Representative images of EdU incorporation (red) into MPCs *in vitro*. Nuclei are visualized with DAPI (blue). (e) YV MPCs, (f) OV MPCs, (g) YS MPCs, and (h) OS MPCs. (i) Quantification of percent EdU+ MPCs. *p < 0.05 between young and old for an individual treatment group (YV vs. OV; YS vs. OS). ^*p* < 0.05 between vehicle and senolytics for a given age group (YV vs. YS; OV vs. OS). *n* = 12–14/group for PBS‐injected mice. *n* = 5–6/group for 7‐ and 28‐day BaCl_2_‐injected mice. For *in vitro* experiments, *n* = 3 technical replicates per group. Scale bar for Pax7 staining equals 50 μm. Scale bar for MPC experiments equals 20 μm

Satellite cells are essential for muscle regeneration (Fry et al., 2015; Relaix & Zammit, 2012) and are lower with aging (Dungan et al., 2020; Shefer et al., 2006, 2010), so we asked whether changes in satellite cell abundance potentially contributed to enhanced muscle regeneration in D+Q‐treated old mice. Satellite cell abundance per area was lower in OV compared to YV PBS‐injected mice, and in OV compared to YV mice 7 days following BaCl_2_‐injected muscle (Figure 5a,b,d), with no difference between YV and OV at the 28‐day time point (Figure 5c,d). There was no effect of D+Q on satellite cell abundance in PBS‐injected young mice, whereas D+Q treatment in old PBS‐injected mice resulted in higher satellite cell abundance (Figure 5d). At 7 days following injury, there was a trend for satellite cells to be elevated in OS mice when compared to OV mice (Figure 5d, *p* = 0.09). There were no differences between groups after 28 days (Figure 5d). To determine if the difference in satellite cell abundance early during regeneration could be due to the effect of D+Q directly on satellite cells, we isolated myogenic progenitor cells (MPCs) from young (4 month) and old (24 month) mice and performed a 24‐h proliferation assay with and without a 24‐h pre‐treatment with D+Q using an EdU incorporation assay to assess newly synthesized DNA. There was no significant effect of D+Q on MPC proliferation in cells isolated from young mice (Figure 5e,g,i), whereas proliferation of MPCs from old mice was augmented (Figure 5f,h,i). Thus, we conclude that some of the beneficial effect of D+Q on regeneration in old mice is due to improved satellite cell proliferation.

### 3.7 Effects of D+Q on gene expression in old mice 7 days following injury

**FIGURE 6:**
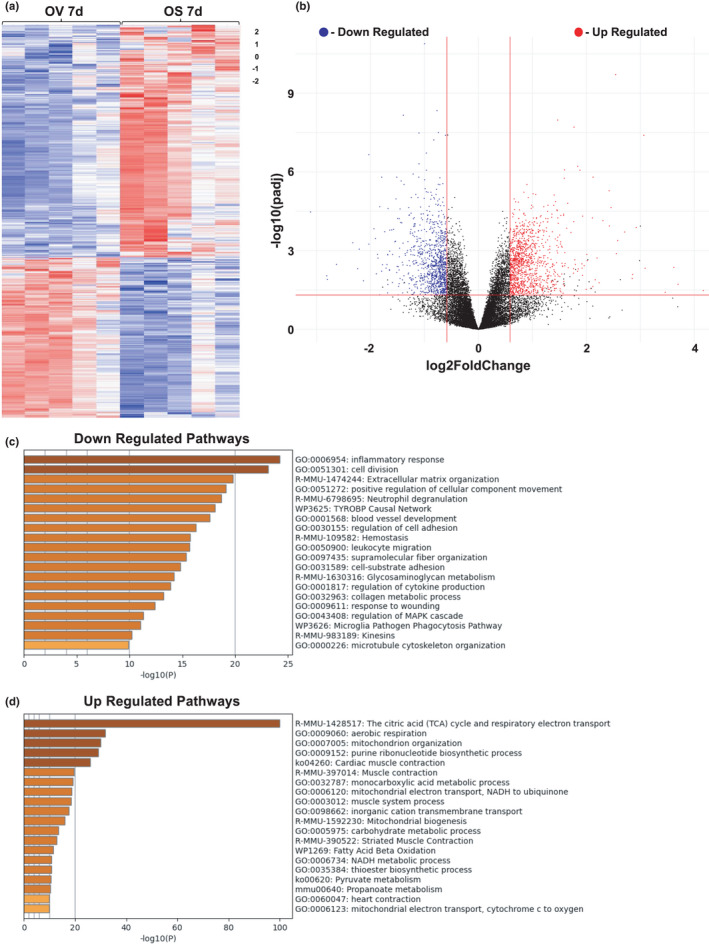
Inflammatory, metabolic, and extracellular matrix pathways are the most affected by the removal of senescent cells. Results of RNA‐seq of muscle from vehicle‐ and senolytic‐treated old mice 7 days following injury displayed by (a) Heat map and (b) Volcano plot. Upregulated (red) and downregulated (blue) genes in OS compared to OV mice are shown. (c) Pathway analysis of downregulated pathways in response to senolytics in old mice. (d) Pathway analysis of upregulated pathways in response to senolytics in old mice. *n* = 5/group

We performed RNA‐seq on whole muscle to identify pathways that were affected by D+Q administration during muscle regeneration in old mice. We focused on the 7‐day time point because of our observation of higher expression of select chemokines in macrophages from old mice relative to young beginning at 7 days post‐injury, and the difference in satellite cells at that time point. In OV vs. OS muscle tissue, there were 4,474 DEGs with an adjusted *p*‐value of <0.05; 2,115 DEGs met our criteria for analysis (see Appendix S1; Figure 6a and File S2). Of the 2115 DEGs, 999 were downregulated and 1116 were upregulated between OV vs. OS mice (Figure 6b). Pathway analysis revealed that the most downregulated pathway in OS relative to OV was inflammatory response (Figure 6c), which corresponded with lower expression of genes associated with the SASP in OS and higher expression of SASP genes in OV. These differences included lower *Ccl2*, *Serpine1*, and *Tlr7* in OS (File S2). There was also reduced expression of anti‐apoptotic genes, specifically, *Survivin* (File S2), which inhibits caspase activity to negatively regulate apoptosis (Shin et al., 2001). Pathway analyses revealed extracellular matrix (ECM) remodeling and negative regulation of angiogenesis pathways were also lower in OS when compared to OV mice (Figure 6c). Genes associated with the induction or maintenance of senescence, such as *Cdkn2a* (p16) and *Myc* (C‐Myc), were also lower following D+Q treatment (File S2). The most upregulated pathways in OS mice included pathways specific to energy production and muscle contraction (File S2; Figure 6d). Specifically, metabolic pathways, such as the TCA cycle and mitochondrial respiration, were among those that were higher in OS mice. In total, D+Q appear to have a beneficial effect on the muscle environment at 7 days post‐BaCl_2_ injury in old mice that likely mediates the significant fiber hypertrophy and improved regeneration observed at 28 days.

## 4 DISCUSSION

In the present study, we examined the contribution of D+Q to early (7 days) and late (28 days) muscle regeneration in both young and old mice. In old mice, D+Q lowered fasting blood glucose, while improving physical function. Following injury, SA β‐Gal+ cells accumulate in muscle from both young and old mice at 7 days and remain elevated in vehicle‐treated old mice 28 days after injury. Vehicle‐treated old mice had a blunted or delayed (Shavlakadze et al., 2010) regenerative response following BaCl_2_ injury compared to young mice, which was rescued by D+Q. Specifically, in old mice, we observed fewer eMyHC+ fibers and more satellite cells early in the regenerative process, along with larger fibers after 28 days. Interestingly, D+Q appeared to blunt muscle regeneration in young mice, as young D+Q‐treated mice have smaller fibers 7‐ and 28 days after BaCl_2_ injury. Further, we show that D+Q affect gene expression in inflammatory and energy producing pathways in old mice, which are essential for muscle regeneration. Our results corroborate and expand upon recent studies showing that following muscle injury, senolytics can effectively lower the senescent cell burden (Chiche et al., 2017), in addition to elevating satellite cell number and causing a shift toward larger fibers (Doan et al., 2020) in old mice. Collectively, these data highlight the potential therapeutic benefits of D+Q following muscle injury in older individuals and provide a word of caution for administration of D+Q in the young.

Senescent cells are shown to accumulate in nearly all tissues; however, senescent cell accumulation in skeletal muscle is extremely low or even undetectable during normal healthy aging (Dungan et al., 2020; Jeyapalan et al., 2007; Sousa‐Victor et al., 2014; Wang et al., 2009). Removal of senescent cells does improve certain measures of physical function (Cai et al., 2020; Doan et al., 2020; Xu et al., 2018), although it does not seem to affect muscle mass (Baker et al., 2016). Dasatinib and quercetin are two senolytic compounds that, when combined (Zhu et al., 2015), have been effective at enhancing renal (Li et al., 2021) and heart (Zhu et al., 2015) function, clearing senescent cells from damaged liver (Chu et al., 2020), improving physical function (Xu et al., 2018) and extending lifespan (Xu et al., 2018). Further, both dasatinib and quercetin modulate the expression of apoptotic/anti‐apoptotic machinery, such as lowering anti‐apoptotic proteins Mcl‐1 (Veldurthy et al., 2008), Bcl‐x_L_ (Veldurthy et al., 2008), and Survivin (Kim et al., 2008), and elevating apoptotic protein p53 (Srivastava et al., 2016). The survivin gene was expressed at lower levels at 7 days post‐injury in our old senolytic‐treated mice when compared to old vehicle‐treated. Very little is known about the contribution of senescent cells to muscle regeneration, although a recent report shows that senescent fibro‐adipogenic progenitor cells are required for adequate regeneration (Saito et al., 2020). This is consistent with work from the Campisi laboratory showing that senescent cells are needed for adequate wound healing (Demaria et al., 2014), leading to the classification of senescent cells being either helper or deleterious (Kirkland & Tchkonia, 2020). We observed a large increase in SA β‐Gal+ cells in both young and old mice 7 days following injury, which are likely infiltrating macrophages based on cell surface expression of CD11b, a pan macrophage marker. Macrophage infiltration is indispensable for normal muscle regeneration, gradually transitioning from a pro‐inflammatory M1 state to a more anti‐inflammatory, reparative M2 state (Saclier et al., 2013; Tidball, 2017). This change in state requires the coordinated effort of various factors, including SA β‐Gal and p16, which are elevated during M1 to M2 polarization and then are reduced following successful transition to an M2 phenoytype (Hall et al., 2016, 2017). RNA‐seq data comparing C_12_FDG+/CD11b+ cells from young and old mice during regeneration suggest these cells are likely becoming senescent or are “stuck” in the pro‐inflammatory state (Hall et al., 2016), only in old muscle. Although the majority (>90%) of SA β‐Gal+ cells were also CD11b+ and likely not senescent 7 days following injury, we cannot discount the potential contribution of the remaining ~10% to defective regeneration in old mice, as a small number of senescent cells have been suggested to have a large impact on tissue homeostasis. SA β‐Gal+ cells may have a key role in the repair of damaged tissue early during regeneration; however, SA β‐Gal+ cells remained elevated in muscle 28 days after injury in old mice, potentially negatively impacting regeneration. D+Q reduced SA β‐Gal+ cells in old mice to that observed in vehicle‐treated young mice, which may directly contribute to enhanced fiber recovery. In young mice, SA β‐Gal+/CD11b+ cells appear to be transitioning to a more M2‐like phenotype, with higher expression of *Adamts1* and *Pdgfb*, factors secreted by M2 macrophages which promote satellite cell activation (Doumit et al., 1993; Du et al., 2017).

Following muscle damage, whether physiologic (i.e., unaccustomed exercise, surgery) or catastrophic (i.e., BaCl_2_, cardiotoxin), satellite cells are an essential component of the regenerative process for skeletal muscle (Fry et al., 2015; Relaix & Zammit, 2012). Older muscle often contains fewer satellite cells (Dungan et al., 2020; Shefer et al., 2006, 2010), which is likely a contributing factor to blunted muscle repair and regrowth with aging. We observed significantly more satellite cells in senolytic‐treated old mice 7 days following BaCl_2_ injection, but no effect in young mice. An inflammatory environment blunts satellite cell proliferation (Perandini et al., 2018; Tierney et al., 2014), and our whole‐muscle RNA‐seq analysis 7 days post‐injury revealed an overall reduction in inflammatory gene expression in muscle from old mice following treatment with D+Q, which may be more conducive to satellite cell proliferation. D+Q may also have direct effects on satellite cells, although we have no evidence that satellite cells are senescent in the 24‐month‐old mice, consistent with the work of others in 42‐month‐old mice (Wang et al., 2009) and old primates (Jeyapalan et al., 2007). It is possible that satellite cells become senescent in very old age, as isolated satellite cells from geriatric mice (>30 months old) appear to senesce *in vitro* more readily than cells from 24‐month‐old mice (Sousa‐Victor et al., 2014). On the other hand, our in vitro experiments suggest that D+Q may directly promote satellite cell proliferation, as MPCs isolated from old mice following treatment with dasatinib and quercetin showed higher EdU incorporation, whereas cells from young mice were not affected. This may be due to effects on MPC metabolism. Whole‐muscle RNA‐seq showed higher expression of genes in glycolytic and oxidative phosphorylation pathways in muscle from old senolytic‐treated mice compared to vehicle 7 days post‐injury, which could have a significant impact, as defects in ATP production delays muscle regeneration (Arneson‐Wissink et al., 2020). Quercetin has been shown to upregulate mitochondrial biogenesis in muscle (Davis et al., 2009; Qiu et al., 2018), which would facilitate energy production required for proliferation. Further, quercetin has antioxidant properties (Zhang et al., 2011), which are maladaptive for muscle growth in young adult rodents (Makanae et al., 2013) and humans (Dutra et al., 2019; Paulsen et al., 2014) due to their inhibitory effect on mTORC1 activation (Ito et al., 2013; Paulsen et al., 2014) but could be beneficial for conditions that exhibit basal hyperactive mTORC1 activation, such as with aging (Dungan et al., 2016; Joseph et al., 2019; Tang et al., 2019).

Quercetin may also directly reduce inflammatory gene expression observed in senolytic‐treated compared to vehicle‐treated old mice 7 days post‐injury. Treatment of U937 differentiated macrophages with quercetin reduced the expression of cytokines, TNF and IL‐1β, and chemokine, MCP‐1 (Overman et al., 2011), factors that were higher in SA β‐Gal+ macrophages from old mice compared to young 14 days following injury. A similar observation was made in Raw 264.7‐derived M1 macrophages, where quercetin reduced TNF, IL‐1β, and MCP‐1 expression (Kim and Park, 2016; Li et al., 2018). Thus, in addition to its antioxidant function and beneficial effects on mitochondrial biogenesis, quercetin may reduce whole‐muscle inflammation, without a concomitant reduction in SA β‐Gal+ cells, early in the regenerative process.

The underlying mechanisms leading infiltrating SA β‐Gal+ macrophages to become senescent following injury in muscle from old mice are currently unknown, but the aged muscle environment is likely a contributor. The ECM in injured muscle from old mice may influence macrophage phenotype (Sicari et al., 2014). RNA‐seq comparing OS vs. OV muscle showed lower expression of a host of ECM genes, including 11 collagen genes, 3 integrin genes, and *Fn1* (fibronectin). Changes in the ECM may promote senescence and a SASP in macrophages in muscle from old mice.

Post‐mitotic senescent cells have gained more traction in recent years as contributors to tissue aging and disease (Sapieha & Mallette, 2018); in brain, senescent neurons express high levels of p21 and contribute to the SASP (Jurk et al., 2012). Basal expression of key senescence marker, p21, is low in skeletal muscle of adult mice relative to other tissues, with little‐to‐no change by 27+ months of age (Yousefzadeh et al., 2020). However, when assessed via single nuclear RNA‐seq, there is a small population of myonuclei from older individuals that express a relatively high amount of p21 (Perez et al., 2021). Further, previous work from our lab shows that human primary myotubes can express high levels of inflammatory cytokines in response to environmental stimuli (Varma et al., 2009). Thus, with aging, damaged muscle fibers could secrete factors that influence macrophages to become senescent. The clearance of senescent myonuclei following D+Q treatment could explain improvements in physical function, without changes in muscle mass or senescent cell burden.

In addition to improvements in muscle regeneration with aging, D+Q improved indices of physical function (rotor rod, balance beam) and whole‐body metabolism (fasting blood glucose). Others have reported similar improvements in physical function following dasatinib + quercetin treatment (Xu et al., 2018), which, considering there is little difference in SA β‐Gal+ cell abundance in resting, uninjured muscle from young and old mice, is potentially due to improvements in muscle proprioceptors and/or the neuromuscular junction. Improvements in glucose uptake following dasatinib + quercetin treatment have been reported during diet‐induced obesity, which was attributed to a reduction of senescent cells in adipose tissue (Palmer et al., 2019). As blood glucose is moderated by multiple tissues (pancreas, liver, skeletal muscle, adipose, etc.) it is likely a concerted effort between tissues that mediates the improvements in blood glucose we observed in old mice.

Together, our data show that clearance of senescent cells is beneficial for muscle regeneration, but only in old mice. Most of the SA β‐Gal+ cells in muscle from old mice following injury are CD11b+ macrophages, whereas in young mice, macrophages transition to a reparative phenotype to facilitate muscle regeneration (Jensen et al., 2020). By 14 days post‐injury, macrophages in adult muscle express factors that facilitate satellite cell function and muscle fiber growth, whereas in old mice they express SASP markers. In addition to reducing the abundance of senescent cells in muscle, senolytics, particularly quercetin, may directly promote satellite cell proliferation to facilitate regeneration. Senolytics effectively reduce senescent cell burden, increase satellite cell abundance, reduce inflammatory gene expression, alter expression of ECM genes, and increase expression of genes in muscle metabolic pathways which may collectively augment muscle fiber regeneration, ultimately leading to increased fiber size and improved physical function. There are still many unknowns about the relationship among senescent cells, skeletal muscle regeneration, and sarcopenia, requiring further study.

## CONFLICT OF INTEREST

Z.J.T., Z.L., A.F., and M.F. are employees of Boehringer Ingelheim Pharmaceuticals, Inc. All other authors have no financial interests.

## AUTHOR CONTRIBUTIONS

C.M.D, A.F., M.F., and C.A.P. designed the study. C.M.D., Z.H., C.Z., K.A.M., G.L.N., C.B., and D.A.E. performed the experiments. Z.J.T. and Z.L. performed the RNA‐seq analysis. C.M.D., K.A.M., C.S.F., A.F., M.F., and C.A.P. prepared the manuscript. All authors reviewed the manuscript.

## Supporting information

Fig S1‐S4

Tab S1

App S1

File S1

File S2

## Data Availability

The data that support the findings of this study are available in the supplementary material of this article.
