# Supplementary material for: Deletion of SA β‐Gal+ cells using senolytics improves muscle regeneration in old mice
Source: Aging Cell. 2021 Dec 13;21(1):e13528. doi: 10.1111/acel.13528 (PMC8761017; doi:10.1111/acel.13528)
Supplement: Supplementary file 1 — Fig S1‐S4 [file ACEL-21-e13528-s003.pptx]

## Slide 1
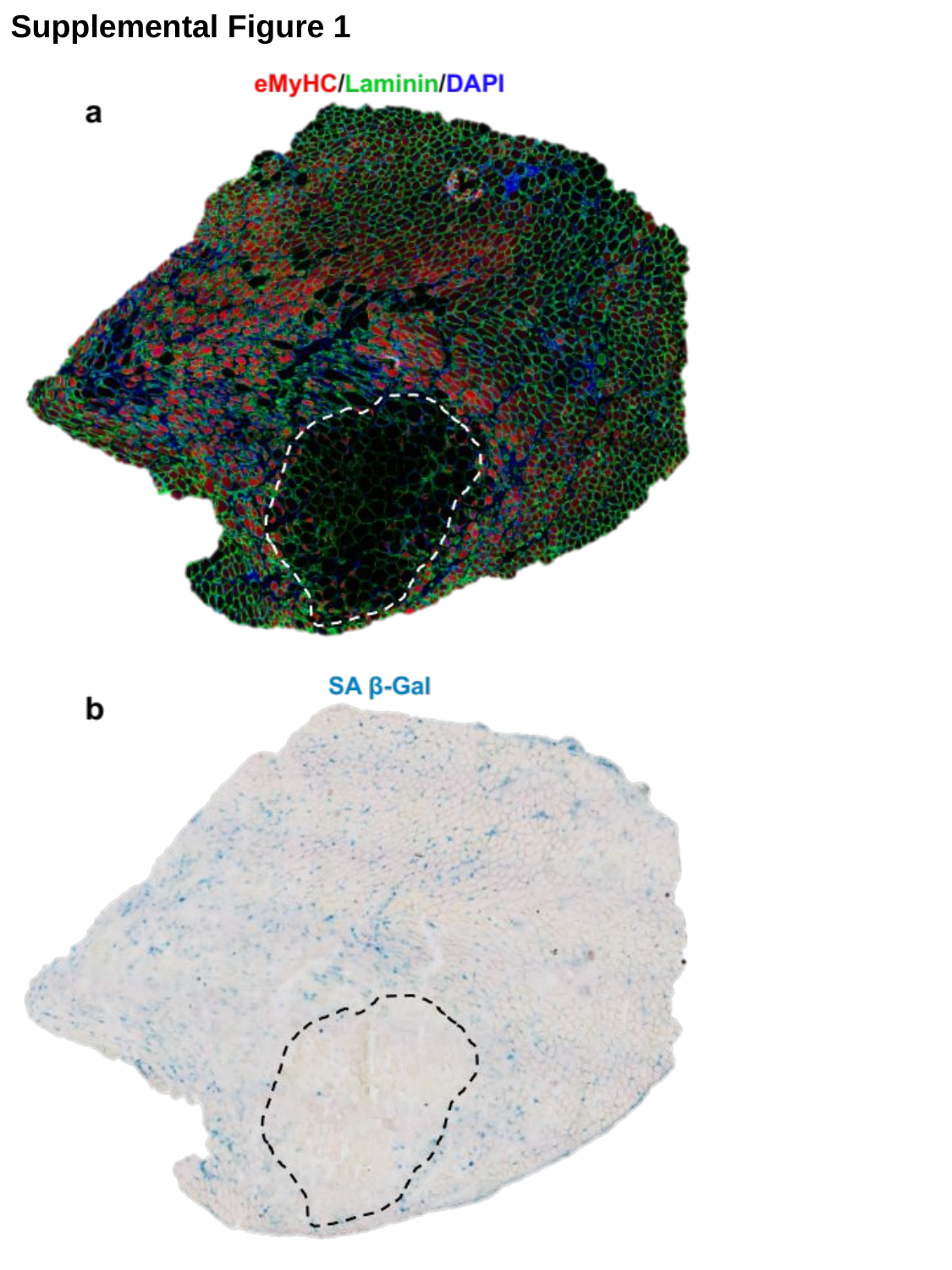

Supplemental Figure 1

## Slide 2
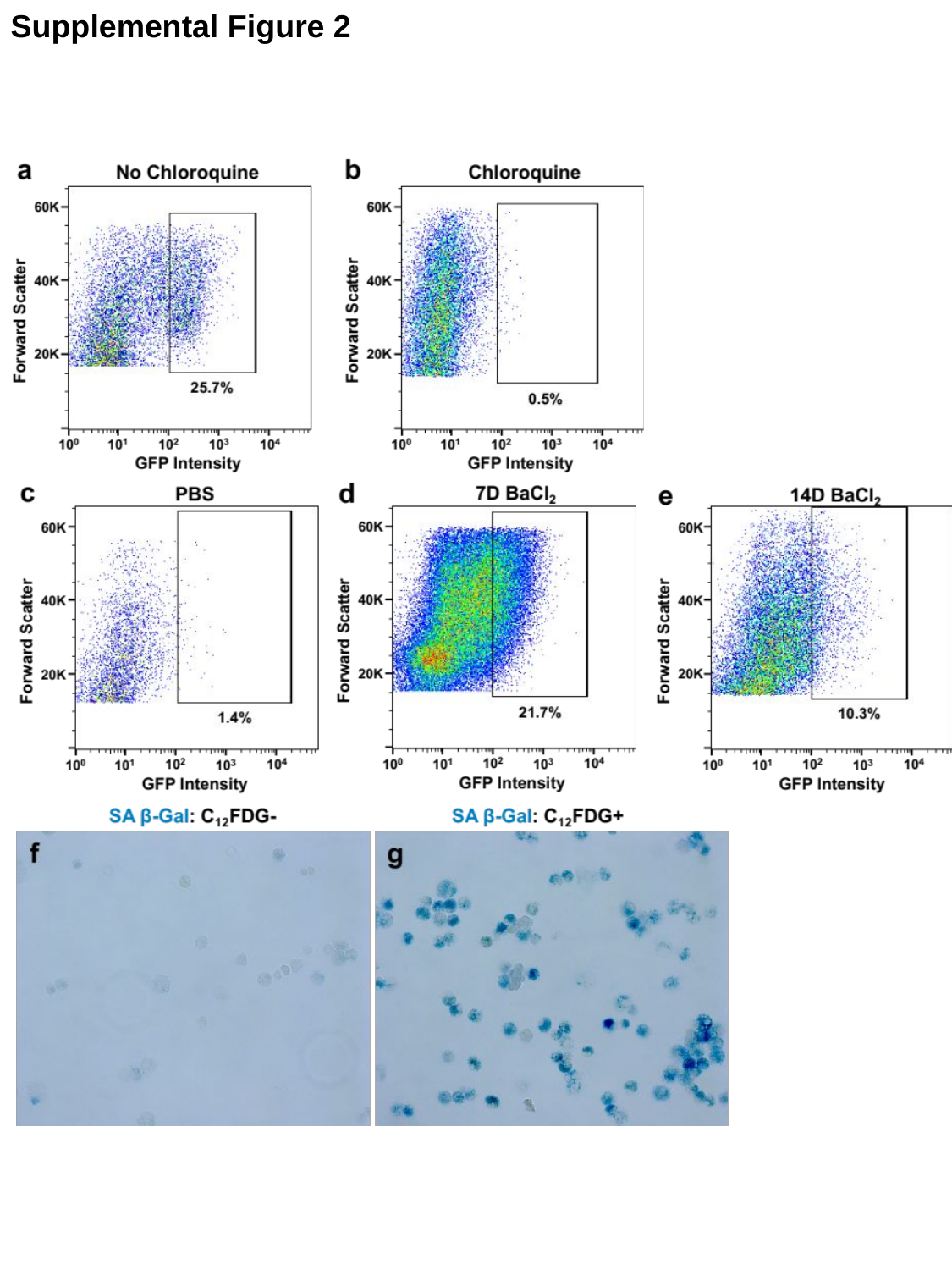

Supplemental Figure 2

## Slide 3
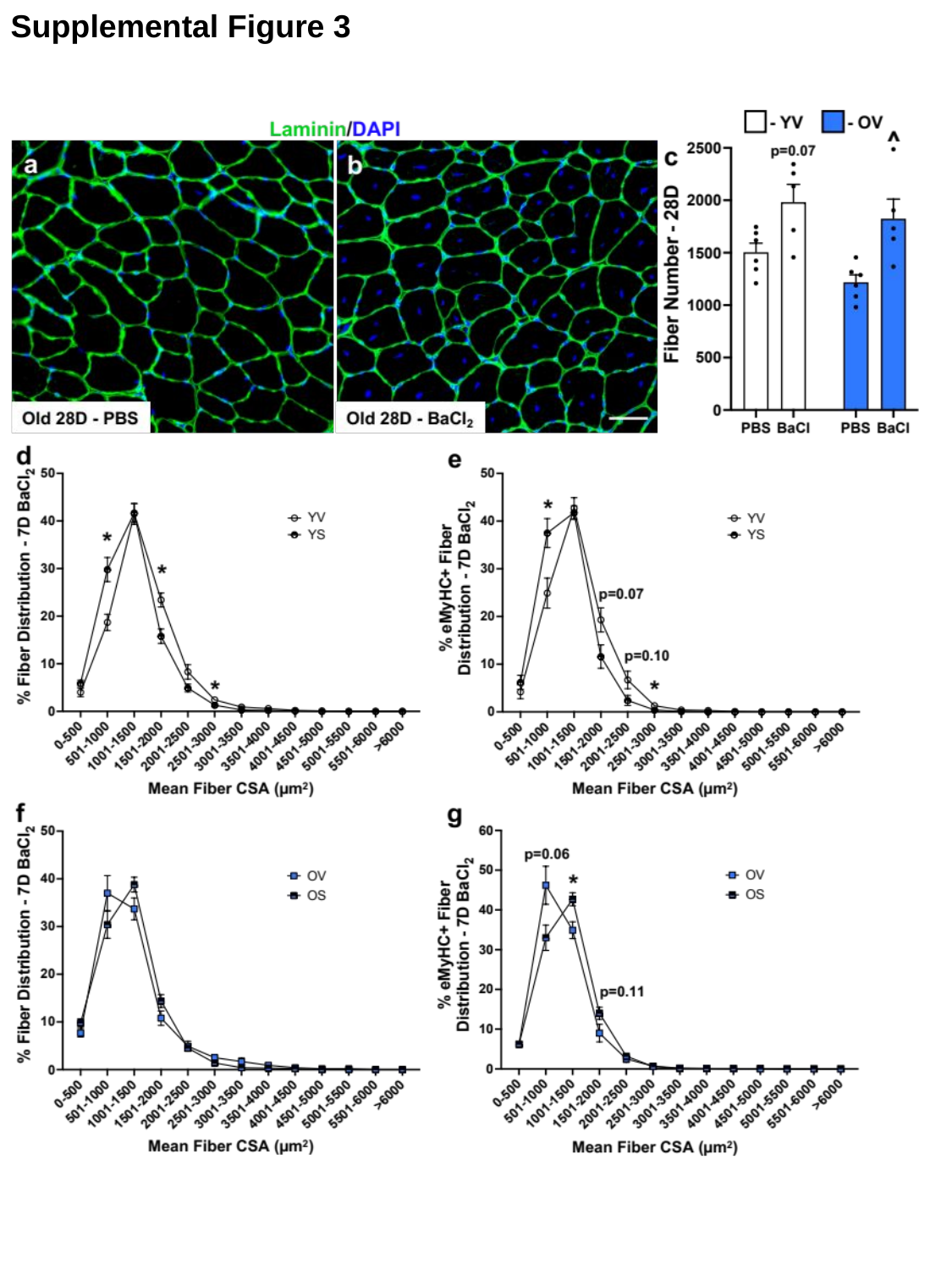

Supplemental Figure 3

## Slide 4
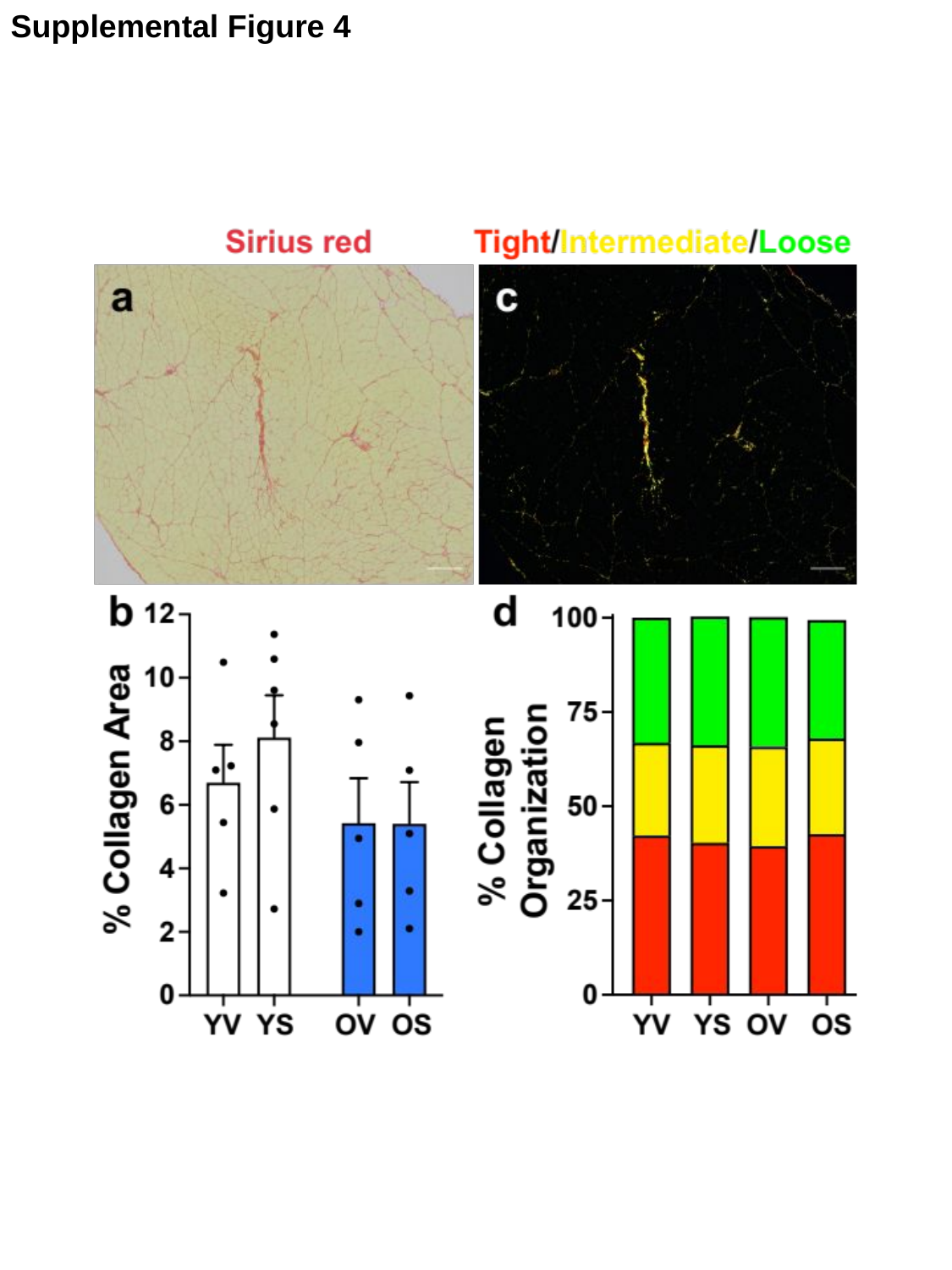

Supplemental Figure 4
