# Supplementary material for: Deletion of SA β‐Gal+ cells using senolytics improves muscle regeneration in old mice
Source: Aging Cell. 2021 Dec 13;21(1):e13528. doi: 10.1111/acel.13528 (PMC8761017; doi:10.1111/acel.13528)
Supplement: Supplementary file 2 — Tab S1 [file ACEL-21-e13528-s001.pptx]

## Slide 1
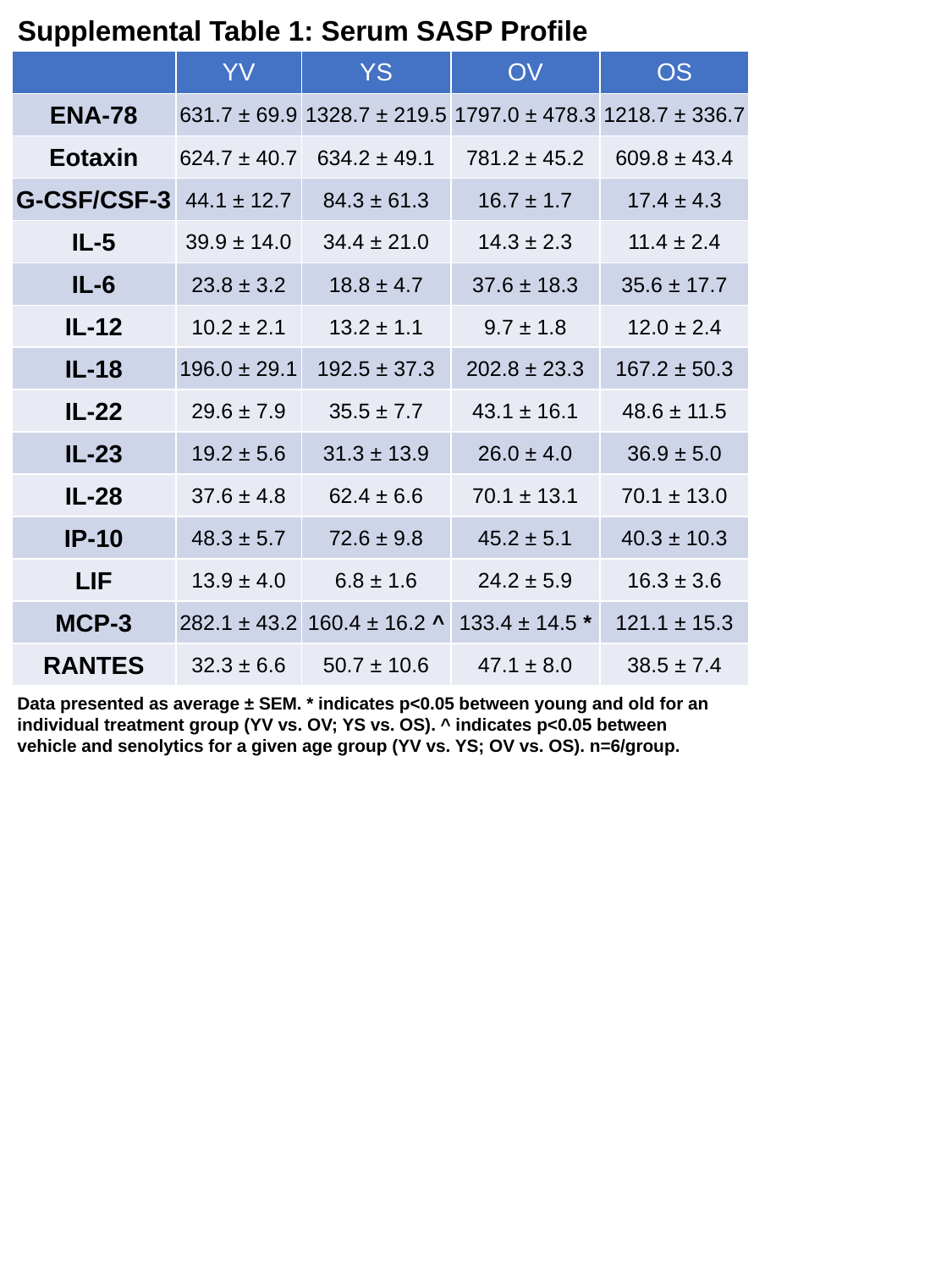

Supplemental Table 1: Serum SASP Profile
| | YV | YS | OV | OS |
| --- | --- | --- | --- | --- |
| ENA-78 | 631.7 ± 69.9 | 1328.7 ± 219.5 | 1797.0 ± 478.3 | 1218.7 ± 336.7 |
| Eotaxin | 624.7 ± 40.7 | 634.2 ± 49.1 | 781.2 ± 45.2 | 609.8 ± 43.4 |
| G-CSF/CSF-3 | 44.1 ± 12.7 | 84.3 ± 61.3 | 16.7 ± 1.7 | 17.4 ± 4.3 |
| IL-5 | 39.9 ± 14.0 | 34.4 ± 21.0 | 14.3 ± 2.3 | 11.4 ± 2.4 |
| IL-6 | 23.8 ± 3.2 | 18.8 ± 4.7 | 37.6 ± 18.3 | 35.6 ± 17.7 |
| IL-12 | 10.2 ± 2.1 | 13.2 ± 1.1 | 9.7 ± 1.8 | 12.0 ± 2.4 |
| IL-18 | 196.0 ± 29.1 | 192.5 ± 37.3 | 202.8 ± 23.3 | 167.2 ± 50.3 |
| IL-22 | 29.6 ± 7.9 | 35.5 ± 7.7 | 43.1 ± 16.1 | 48.6 ± 11.5 |
| IL-23 | 19.2 ± 5.6 | 31.3 ± 13.9 | 26.0 ± 4.0 | 36.9 ± 5.0 |
| IL-28 | 37.6 ± 4.8 | 62.4 ± 6.6 | 70.1 ± 13.1 | 70.1 ± 13.0 |
| IP-10 | 48.3 ± 5.7 | 72.6 ± 9.8 | 45.2 ± 5.1 | 40.3 ± 10.3 |
| LIF | 13.9 ± 4.0 | 6.8 ± 1.6 | 24.2 ± 5.9 | 16.3 ± 3.6 |
| MCP-3 | 282.1 ± 43.2 | 160.4 ± 16.2 ^ | 133.4 ± 14.5 \* | 121.1 ± 15.3 |
| RANTES | 32.3 ± 6.6 | 50.7 ± 10.6 | 47.1 ± 8.0 | 38.5 ± 7.4 |
Data presented as average ± SEM. * indicates p<0.05 between young and old for an individual treatment group (YV vs. OV; YS vs. OS). ^ indicates p<0.05 between vehicle and senolytics for a given age group (YV vs. YS; OV vs. OS). n=6/group.
