# Supplementary material for: Deletion of SA β‐Gal+ cells using senolytics improves muscle regeneration in old mice
Source: Aging Cell. 2021 Dec 13;21(1):e13528. doi: 10.1111/acel.13528 (PMC8761017; doi:10.1111/acel.13528)
Supplement: Supplementary file 3 — App S1 [file ACEL-21-e13528-s002.docx]

**Sirius Red Staining and Analysis**

Sirius red staining for fibrous collagen was performed as previously described^1^**.** Muscle sections were fixed in Bouin’s solution (#15990-10, Electron Microscopy Sciences, Hatfield, PA) in 56°C water bath for 1 hour. Slides were washed in PBS and incubated in Sirius Red solution (ab150681, Abcam, Cambridge, United Kingdom; 0.1% in saturated picric acid) at room temperature for 2 hours. Slides were washed in 0.5% acetic acid, followed by dehydration in 95% and 100% ethanol and equilibration in xylenes. Lastly, slides were mounted in xylene-based mounting media. Sirius red staining was imaged and quantified using Image J Fiji software as previously described^2^**. The area of Sirius red collagen is reported as a percentage of the total muscle area. Additionally, Sirius red was imaged under polarized light to denote tightly packed (red), intermediate (yellow), and loosely packed (green) collagen fibrils^3^, and quantification was performed using the color pixel counter plugin with Image J Fiji software to count red, green, and yellow pixels relative to total pixel count of the whole muscle section.**

**FACS Isolation and Maintenance of Myogenic Progenitor Cells (MPCs)**

Myogenic progenitor cells were isolated as previously described by our laboratory via FACS^4,5^. Briefly, muscles of the mouse hindlimb were processed as described above. Single cell suspensions were incubated with antibodies against Vcam (105704, BioLegend), Cd31 (11-0311-82, Invitrogen), Cd45 (103108, BioLegend), and Sca1 (557405, BD Pharmingen, Franklin Lakes, NJ) at 4°C; a biotinylated secondary antibody was used for Vcam. Cells were pelleted at 500 x g, re-suspended, and sorted using an iCyt FACS machine (Sony Biotechnology). Appropriate isotype-specific controls were used to validate antibody specificity, and dead cells were gated out using forward/side scatter profiles and propidium iodide. Satellite cells were identified as Vcam+/Cd31-/Cd45-/Sca1-. MPCs were maintained in 20% O_2_ and 5% CO_2_ and expanded in growth media consisting of Ham’s F10+15% normal horse serum+1% penicillin/streptomycin supplemented with basic fibroblast growth factor (5 ng/ml, Millipore, St. Louis, MO) on plates coated with ECM gel diluted 1:100 (Sigma-Aldrich).

**Senescence-associated β-Galactosidase (SA β-Gal) Staining of** **C_12_FDG+ and C_12_FDG- cells**

For in vitro experiments, cells were fixed in 0.5% glutaraldehyde for 5 minutes at room temperature, washed in PBS, and then incubated in SA β-Gal staining solution at 37°C in a dark hybridization oven for 16 hours. Afterwards, slides were washed in PBS for 3 x 5 minutes, post-fixed in 0.5% glutaraldehyde for 10 minutes, washed in PBS, and then cover slipped using PBS and glycerol (1:1).

**RNA Sequencing**

Two µg (bulk mRNA sequencing) and 250 ng (low-input mRNA) of high-quality RNA was sent to Novogene (Beijing, China) for mRNA sequencing. Low-input RNA sequencing was performed using a modified protocol for small quantities of RNA with additional PCR cycles relative to bulk RNA sequencing. Library preparation and sequencing were carried out as paired-end reads of 150 bps. Sequencing quality was assessed with FastQC, Picard, and MultiQC. For each sample, adapters and low-quality base pairs (<=10) were trimmed to the right by BBDuk with K=13 and mink=5. All shorter reads (<20 base pairs) were also removed. Cleaned reads were aligned to the Genome Reference Consortium Mouse Build 38 (GRCm38) with STAR aligner (STAR_2.5.2b)^6^. Per sample gene expression profiles were estimated by feature Counts (v1.5.1)^7^ using the Ensembl gene annotation (version GRCm38.86). Both inter- and intra-group variability and outlier detection were carefully examined using both principal component analysis (PCA) and sample correlation analysis. Differential gene expression analysis was performed using DESeq2 (v1.26.0)^8^. Significant differentially expressed genes (DEGs) were identified with adjust *P* value of <= 5%, fold-change of 1.5, and base mean value of >=10 using Partek® Flow® Genomics Software (St. Louis, MO). Metascape was applied to find enriched pathways using significant differentially expressed up-regulated and down-regulated genes separately^9^.

**Multiplex ELISA on serum**

To isolate serum, whole blood was collected in 1.5 mL tubes and allowed to sit at room temperature for 30 minutes. Tubes were then spun at 3,000 x g for 15 minutes, serum was collected, and frozen at -80°C. Serum cytokines from injection-naive mice were assessed in triplicate using the Cytokine & Chemokine 36-Plex Mouse ProcartaPlex™ Panel 1A (EPX360-26092-901, Invitrogen) according to the manufacturer’s instructions, and the plate was read on a Luminex FlexMap 3D system (Luminex, Austin, TX). The average minimum threshold was set at 10.

1. Fry CS, Kirby TJ, Kosmac K, McCarthy JJ, Peterson CA. Myogenic Progenitor Cells Control Extracellular Matrix Production by Fibroblasts during Skeletal Muscle Hypertrophy. *Cell Stem Cell.* 2017;20(1):56-69.

2. Fry CS, Johnson DL, Ireland ML, Noehren B. ACL injury reduces satellite cell abundance and promotes fibrogenic cell expansion within skeletal muscle. *J Orthop Res.* 2017;35(9):1876-1885.

3. Smith LR, Barton ER. Collagen content does not alter the passive mechanical properties of fibrotic skeletal muscle in mdx mice. *Am J Physiol Cell Physiol.* 2014;306(10):C889-898.

4. Murach KA, Peck BD, Policastro RA, et al. Early satellite cell communication creates a permissive environment for long-term muscle growth. *iScience.* 2021;24(4):102372.

5. Murach KA, Vechetti IJ, Jr., Van Pelt DW, et al. Fusion-Independent Satellite Cell Communication to Muscle Fibers During Load-Induced Hypertrophy. *Function (Oxf).* 2020;1(1):zqaa009.

6. Dobin A, Davis CA, Schlesinger F, et al. STAR: ultrafast universal RNA-seq aligner. *Bioinformatics.* 2013;29(1):15-21.

7. Liao Y, Smyth GK, Shi W. featureCounts: an efficient general purpose program for assigning sequence reads to genomic features. *Bioinformatics.* 2014;30(7):923-930.

8. Love MI, Huber W, Anders S. Moderated estimation of fold change and dispersion for RNA-seq data with DESeq2. *Genome Biol.* 2014;15(12):550.

9. Zhou Y, Zhou B, Pache L, et al. Metascape provides a biologist-oriented resource for the analysis of systems-level datasets. *Nature communications.* 2019;10(1):1523.
